# "Primary" nocardial brain abscess in a renal transplant patient

**DOI:** 10.1186/s13104-015-1697-4

**Published:** 2015-11-23

**Authors:** Ranga Migara Weerakkody, Dhammika Randula Palangasinghe, Saman Wadanambi, Eranga Sanjeewa Wijewikrama

**Affiliations:** University Medical Unit, National Hospital of Sri Lanka, Regent Street, Colombo, 9 Sri Lanka; Department of Neurosurgery, National Hospital of Sri Lanka, Regent Street, Colombo, 9 Sri Lanka; Department of Medicine, Faculty of Medicine, University of Colombo, Kynsey Road, Colombo, 8 Sri Lanka

**Keywords:** Cerebral abscess, *Nocardia*, Renal transplantation, Short antibiotic course

## Abstract

**Background:**

Intracranial abscesses are rare among transplant recipients, and *Nocardia* is responsible for less than 2 % of them. Nocardiosis, a chronic infection and is difficult to treat. Primary infection involves lungs and eventually disseminates. Primary nocardial abscesses are rare and we report a case from Sri Lanka.

**Case presentation:**

A 38 year old Sri Lankan, who has received his 2nd ABO matched live donor transplantation, which was complicated with perinephric hematoma and massive transfusion syndrome. He presented with fever, worsening headache and papilledema. An urgent magnetic resonance image (MRI) showed an occipital abscess with midline shift. Craniotomy and drainage followed by 3 week course of imipenem and levofloxacin, which rendered him symptom free. After 12 months he has stayed recurrence free. Imaging and bacteriology of the respiratory tract failed to demonstrate *Nocardia* infection.

**Conclusion:**

Isolated (Primary) nocardial brain abscess are rare, and have an excellent response to medical therapy. We achieved a good response from a relatively short course of antibiotics (not using sulfonamides, due to allergy), where long courses of antibiotic had been the norm.

## Background

Cerebral abscess are rare among the renal transplant (RT) patients with an incidence of 3.6 per 1000 cases/year [1]. *Nocardia* is only responsible for 12 % of above [2], but strongly associated with immunosuppressed state. *Nocardiae* are gram positive, partially acid fast, and filamentous bacilli found in soil [3]. The infection is chronic, usually originate in the lungs, and spread to other organs. Isolated (or “primary”) cerebral abscess, without skin or lung involvement is an exceedingly rare finding [3, 4]. We are reporting such a case occurring in a RT patient.

## Case presentation

A 38 year old Sri Lankan businessman from central metropolis of Colombo—who has received his second RT in January, 2013—presented with headache, confusion and fever of 38 °C in February 2014. He had a past history of rapidly progressive glomerulonephritis in February 2007, and was treated with high dose prednisolone and six pulses of cyclophosphamide to no avail. He developed end stage renal failure (ESRD), was on hemodialysis for 14 months and received his first ABO matched, live related RT in July 2009. Eighteen months after transplantation in May 2011, he developed severe acute antibody and cell mediated rejection and was treated with plasmapheresis, intravenous immunoglobulin and rabbit anti-thymocyte globulin. However he did not improve and his graft failed and developed ESRD by July 2011, and re-commenced on dialysis. His second RT was an ABO matched, live non related donor transplant. He was induced with 75 mg of rabbit anti-thymocyte globulin and was on maintenance immunosuppression with tacrolimus and mycophenolate. Immediate post of period was complicated by a massive perinephric hematoma, which needed evacuation and drainage. As a result he developed massive transfusion syndrome, and was resuscitated and managed accordingly. After initial stormy period, he had a satisfactory graft function (serum creatinine 121 μmol/l), and event free post RT period. He was allergic to sulfur, and has refused pentamidine prophylaxis for pneumocyctis infection. He was CMV IgG positive since his first transplant and had two 90 day courses of valganciclovir prophylaxis dose (450 mg daily) following each RT. He never had CMV IgM positivity or a significant viral load throughout both of his RTs.

His current illness started with a gradually worsening headache, and fever. He did not have photophobia, neck pain, skin nodules or rashes. He did not give a preceding history of lower respiratory tract illness or cough. On examination he was febrile but alert, and demonstrated no neck stiffness. No lymphadenopathy or organomegaly was noticed. Central nervous system examination was normal other than for bilateral papilledema. Cardiovascular and respiratory systems were clinically normal on examination.

**Fig. 1:**
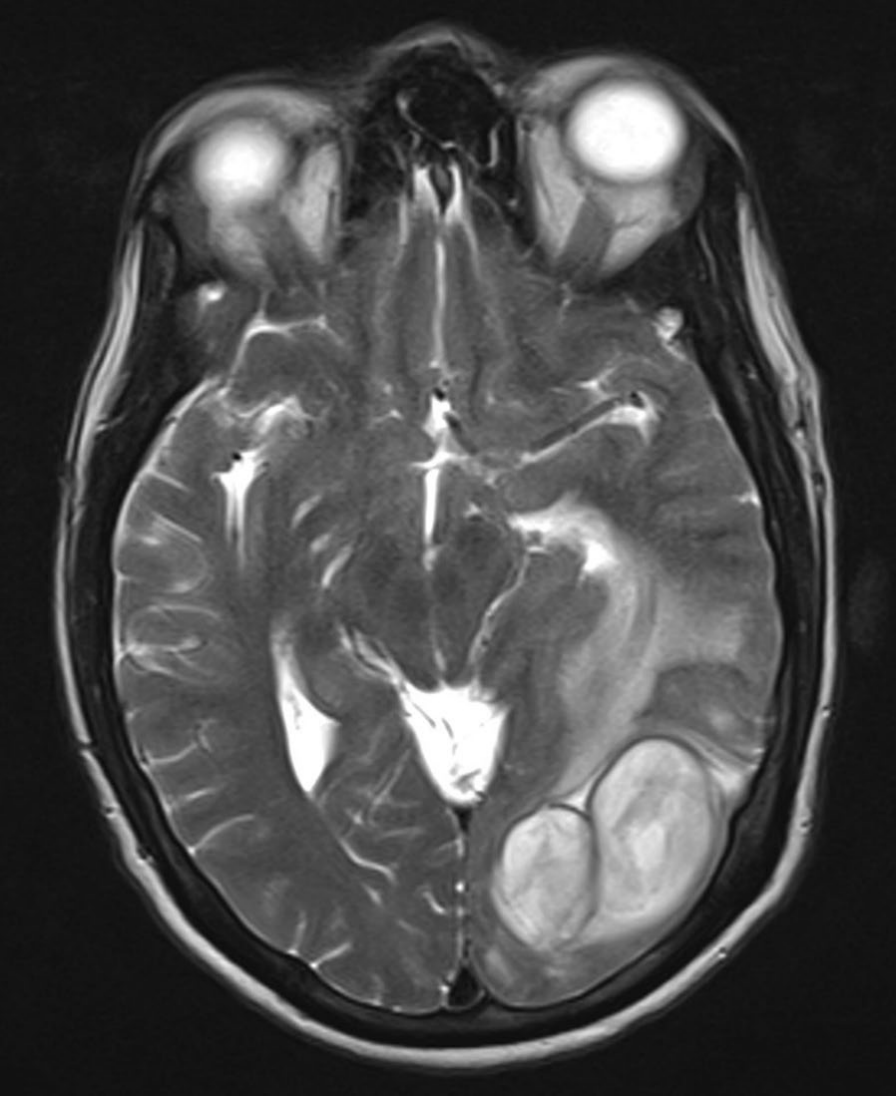
Brain MRI. MRI scan of the brain showing two closely related cerebral abscesses in the left occipital region and resulting peri-lesional edema and the mid-line shift

**Fig. 2:**
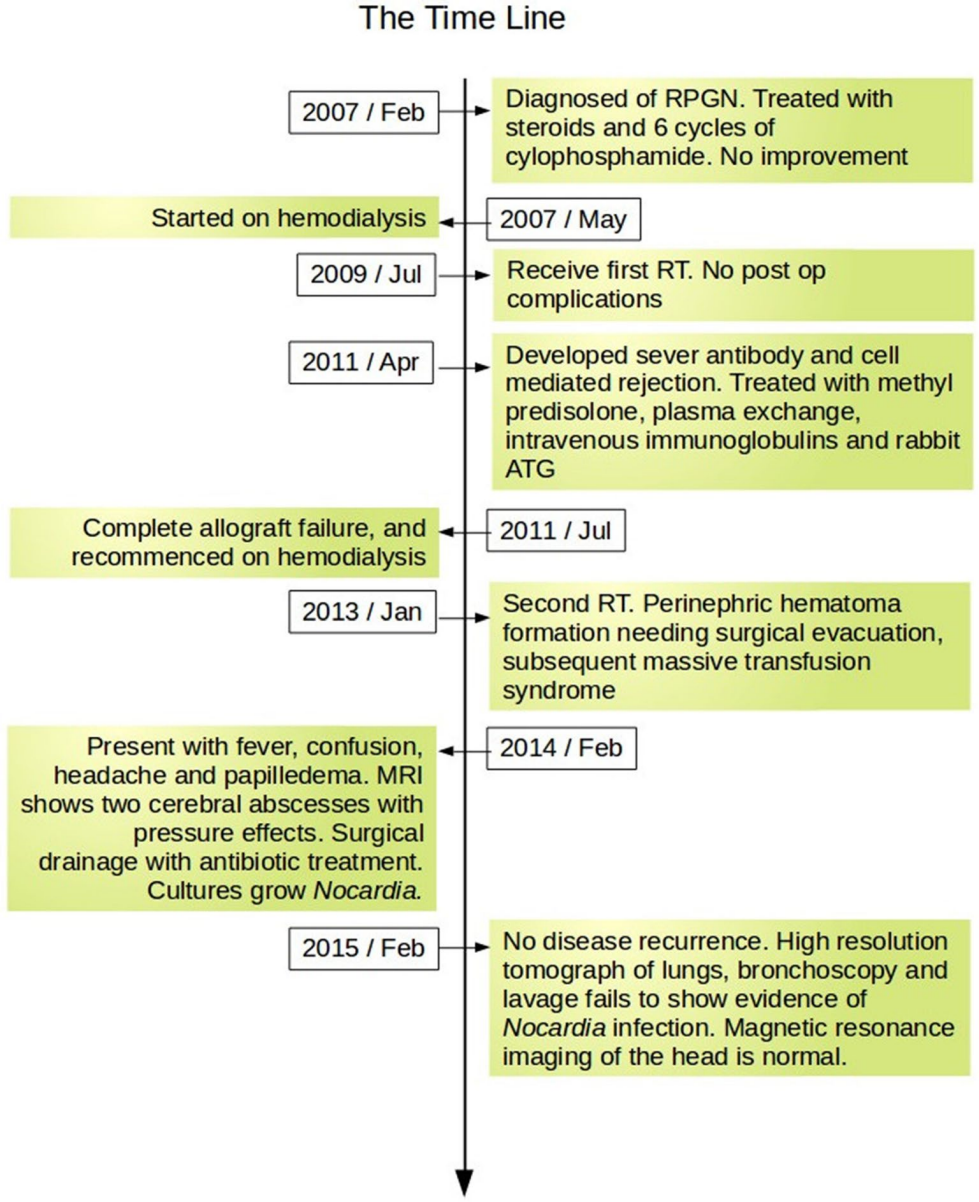
The time line of the case discussed

With worsening symptoms, an urgent non contrast computed tomography of the head was performed. It showed two hypodense areas with largest measuring 5 × 4 × 3 cm in size, located in the occipital pole within the grey matter, with surrounding cerebral edema. Urgent gadolinium enhanced MRI was performed and it showed two large abscesses in the occipital area with peri-lesion edema and midline shift (Fig. 1). Blood investigations demonstrated neutrophil leukocytosis (12.5k/μl), elevated C-reactive protein (122 μg/dl) and mildly elevated serum creatinine (129 μmol/l). Two abscesses were surgically drained via burr hole aspiration, followed by saline washout. The purulent material drained cultured *Nocardia* spp, with characteristic morphological pattern. We started intravenous imipenem and intravenous levofloxacin, and continued them according to the susceptibility in the culture results. Chest X rays and high resolution CT scans failed to show pulmonary nodules suggestive of nocardiosis. He became symptom free after 21 day course of antibiotics with serum investigations returning to basal levels. Other than for a mild elevation of serum creatinine, his graft function remained stable throughout the treatment period. Twelve months after discharge he has not shown any recurrence of the disease. His follow up magnetic resonance image (MRI) imaging was normal. He was assessed for lung nocardiosis with a repeat HRCT, and a bronchoscopy and lavage, but both tests failed to demonstrate any abnormalities. Figure 2 graphically shows the time line.

## Discussion

Nocardiosis is a rare infection, which predominately affects lungs, brain, or the skin, and is an infection of the immunosuppressed host [5]. Lung is the primary focus, and it spreads to other organs—notably brain and skin—through blood. Spread to other organs such as kidneys, the joints, the heart, the eyes, and the bones have been reported [3]. *Nocardia* is fastidious organism found in soil, and acquired via inhalation. *Nocardia asteroides* is responsible for the 90 % of infections while *N. brasiliensis* and *N. caviae* accounts for the majority of the rest [6, 7].

The taxonomic history of the genus *Nocardia* is fraught with confusion and controversy. The genus *N. asteroides* contains many species and referred to as *N. asteroids* complex in modern literature. *Nocardia* species demonstrate variety of drug susceptibility patterns which could be used for species recognition. Six patterns have been described in literature. New patterns have been described since then, which are associated with newly found species. The susceptibility to imipenem and fluroquinolones, and resistance to ceftriaxone, places the isolate from our patient in sensitivity pattern V, which corresponds to *N. farcinia* [8].

Receipt of high-dose steroids, history of cytomegalovirus disease, and high levels of calcineurin inhibitors are independent risk factors for *Nocardia* infection in solid organ transplant recipients [9]. Lung and skin are the primary sites of infection in most of the described cases of nocardiosis. Nocardial cerebral abscess without evidence of involvement of other organs is rare [3, 4]. The pathogenesis of an isolated cerebral abscess is unclear, but blood borne spread from infested (but un-infected) lung is one explanation. Thorough skin examination imaging and microbiological studies of the respiratory tract failed to produce evidence of *Nocardia* infection. Overall mortality of nocardial abscesses is 31–77 % [3, 10], and reported “primary” nocardial brain abscesses in solid organ transplants [1, 3, 4] were treated with 100 % success, which may point towards a different pathogenesis. Trimethoprime–sulfamethoxole is described as an integral part of the treatment, but this case report shows favorable outcomes can be achieved without using it. Cephalosporins, carbepenems, fluoroquinolones, linezolid and doxycycline had been documented used to treat nocardiosis [1, 9, 11, 12]. The treatment is usually continued for 3–12 months [9, 13], but in this case we achieved remission, and free of recurrence at least at 12 months of follow up, by only using 3 week course of imipenem and levofloxacin, which was the one of the shortest course of antibiotics used in any of the studies. This may have been possible because of early surgical intervention which was highly successful, due to the favorable site of the lesions.

## Conclusion

Primary nocardial cerebral abscess is rare in transplanted patients and has a very good prognosis compared to disseminated disease. Long courses of antibiotics are recommended, but this case shows complete recovery is possible with even 3 weeks of antibiotics, provided the abscesses could be successfully drained. Trimethoprim–sulfamethaxole is not essential in the treatment as shown in this case.

## Consent

Written informed consent was obtained from the patient for publication of this case report and any accompanying images.

## Abbreviations

MRI: magnetic resonance image
ESRD: end stage renal failure
RT: renal transplant
